# Testosterone abuse in Brazil: a growing public health challenge

**DOI:** 10.1016/j.clinsp.2026.101036

**Published:** 2026-07-09

**Authors:** Adriano Fregonesi, Bruno Chiesa Gouveia Nascimento, José Maria Soares Junior, Ana Cláudia Latronico, Carmita Helena Najjar Abdo, Lucas Vasconcelos Sanvido, Archimedes Nardozza Junior, Fernando Nestor Facio Junior, José de Bessa Junior, Luiz Otávio Torres, Sidney Glina, Jorge Hallak, Edson Ferreira, Gustavo Maciel, Elaine Maria Frade Costa, Arnaldo José Hernandez, André Pedrinelli, Eduardo Ferriolli, Edmund Chada Baracat, William Carlos Nahas, Cristiano Mendes Gomes

**Affiliations:** a Disciplina de Urologia, Universidade Estadual de Campinas (UNICAMP), Campinas, SP, Brazil; b Disciplina de Urologia, Hospital das Clínicas, Faculdade de Medicina, Universidade de São Paulo (HCFMUSP), São Paulo, SP, Brazil; c Disciplina de Ginecologia, Departamento de Obstetricia e Ginecologia, Hospital das Clínicas, Faculdade de Medicina, Universidade de São Paulo (HCFMUSP), São Paulo, SP, Brazil; d Disciplina de Endocrinologia e Metabologia, Hospital das Clínicas, Faculdade de Medicina, Universidade de São Paulo (HCFMUSP), São Paulo, SP, Brazil; e Instituto PROSEX, Hospital das Clínicas, Faculdade de Medicina, Universidade de São Paulo (HCFMUSP), São Paulo, SP, Brazil; f Disciplina de Urologia, Universidade Federal de São Paulo (UNIFESP), São Paulo, SP, Brazil; g Disciplina de Urologia, Faculdade de Medicina de São José do Rio Preto (FAMERP), São José do Rio Preto, SP, Brazil; h Departamento de Cirurgia, Universidade Estadual de Feira de Santana (UEFS), Feira de Santana, BA, Brazil; i Centro Universitário UniBH, Belo Horizonte, MG, Brazil; j Serviço de Urologia, Faculdade de Medicina do ABC, Santo André, SP, Brazil; k Departamento de Ortopedia e Traumatologia, Faculdade de Medicina, Universidade de São Paulo (FMUSP), São Paulo, SP, Brazil; l Departamento de Clínica Médica, Faculdade de Medicina, Universidade de São Paulo (FMUSP), São Paulo, SP, Brazil; m Faculdade de Medicina, Universidade de São Paulo (FMUSP) e Universidade Nove de Julho (UNINOVE), São Paulo, SP, Brazil

## Introduction

Testosterone, the primary circulating androgen in men, is a vital steroid hormone involved in a broad spectrum of physiological processes, including mood regulation, sexual function, bone and muscle homeostasis, and metabolic processes.^1^^,^^2^ Although testosterone deficiency is a formally recognized medical entity and treatment with testosterone compounds is recommended by several national and international associations in carefully selected cases, the non-medical use and abuse of testosterone and other Anabolic-Androgenic Steroids (AAS) have expanded significantly in recent decades, transcending elite athletics to become a major worldwide concern because of the serious associated health risks.^1^, ^2^, ^3^, ^4^

Pharmacologically, AAS include testosterone itself and its esters, such as testosterone decanoate, propionate, and isocaproate, as well as a variety of synthetic derivatives such as nandrolone, trenbolone, oxandrolone, and drostanolone. These substances exhibit both anabolic properties, promoting muscle growth, and androgenic effects related to the development of male secondary sexual characteristics, although their potency and selectivity vary significantly across different agents.^3^

Recognizing the physiological and clinical relevance of testosterone, an one-day course held in São Paulo, Brazil, convened leading experts from endocrinology, urology, gynecology, geriatrics, psychiatry, sports medicine, and related specialties to provide an update on testosterone science and practice. The scientific program addressed the role of testosterone and other androgens in male and female health, age-related changes, the challenges of diagnosing hypogonadism, cardiovascular risk, fertility, sexual function, and regulatory and ethical issues. Appropriate use of Testosterone Replacement Therapy (TRT) was extensively discussed, emphasizing that testosterone is a legitimate treatment for men with confirmed hypogonadism when used under strict medical supervision and prescribed with the aim of restoring physiological levels. In this context, guideline-based therapy and contemporary safety data on medically supervised TRT should not be conflated with the non-medical use of supraphysiologic androgen doses.^4^^,^^5^ The program also included practical roundtable discussions involving complex and controversial scenarios in testosterone therapy, such as men with hypogonadism undergoing treatment for prostate cancer. In parallel, the event highlighted the current concerning scenario of androgen abuse. In this editorial, AAS abuse refers to the non-medical, supraphysiologic use of testosterone formulations and other synthetic AAS primarily for appearance and/or performance enhancement. Approved products such as testosterone decanoate, propionate, and isocaproate may be used as legitimate TRT when prescribed at physiological doses in patients with hypogonadism; however, when used non-medically with the aim of achieving supraphysiologic levels, they constitute testosterone abuse.^1^, ^2^, ^3^, ^4^, ^5^, ^6^, ^7^ This distinction between guideline-based TRT and AAS abuse is essential to frame the clinical and public health concerns discussed in the present manuscript.

## Testosterone abuse: user demographics and motivations

Historically confined to elite sports, the contemporary profile of the non-medical AAS user is predominantly composed of non-athlete young or middle-aged men, but use among women and older people is increasingly recognized, particularly in physique- and fitness-oriented contexts.^8^^,^^9^

In Brazil, available data on the indiscriminate use of AAS suggest a pervasive problem, particularly among practitioners of recreational physical activity.^10^ A systematic review indicated that the prevalence of AAS use among practitioners of recreational physical activity is highly variable, ranging from 2.1% to 31.6%, depending on region and sample characteristics.^10^ Studies focused on specific populations have shown high rates, such as 31.6% among physical education students and teachers in Belém do Pará, in the Northern Region of Brazil,^11^ and 19% among bodybuilders attending major gym centers in São Paulo.^12^ Globally, the lifetime prevalence of AAS use has been estimated at 3.3%, with use being far more common among males than females.^8^ This pervasive non-medical use is often referred to in the medical literature as a hidden epidemic.^2^^,^^3^^,^^13^

This pattern aligns with global observations showing that recreational sportspeople and gym clients have particularly high rates of AAS exposure.^8^ The motivation for abuse centers predominantly on achieving aesthetic or image-enhancement goals, such as looking muscular and lean, rather than pursuing competitive athletic performance.^2^^,^^3^ A major underlying psychological factor driving this behavior is muscle dysmorphia, a body image disorder characterized by an obsessive feeling of being insufficiently muscular, which may motivate and perpetuate AAS use.^13^

## Patterns of non-medical testosterone use

Illicit AAS use routinely involves supraphysiologic doses that dramatically exceed replacement therapy levels.^1^^,^^14^^,^^15^ While medically supervised TRT employs physiological dosing, individuals who abuse AAS may consume substantially higher weekly doses and undergo prolonged cycles of use.^1^^,^^14^^,^^15^ In one cohort of male amateur athletes, the median cycle length was 13-weeks, and substantial variation in weekly AAS-equivalent dose was documented.^14^

AAS are characteristically used in structured “cycles”, consisting of intensive use periods spanning several weeks or months, deliberately separated by “off” periods.^1^^,^^14^^,^^15^ Users commonly combine multiple compounds, a practice known as “stacking”, and may also adopt dose escalation strategies. Evidence suggests that prior use is associated with longer and heavier subsequent cycles, indicating progression of exposure over time.^14^^,^^15^ Critically, a substantial minority of users may eventually develop AAS dependence syndrome.^2^^,^^15^^,^^16^

## Sourcing and black-market dynamics

Given the status of AAS as controlled substances, much of the non-medical consumption relies on illicit acquisition routes, often through black-market channels and internet-based access.^15^, ^16^, ^17^ The black market presents substantial and unpredictable risks because of poor quality control. A systematic review found that a considerable proportion of black-market AAS products are counterfeit or substandard, meaning that the declared substance, concentration, or both may be inaccurate.^17^

In Brazil, this problem is compounded by forensic evidence showing counterfeit anabolic products seized by authorities and by a marked increase in the quantity of seized anabolic steroids over time.^18^^,^^19^ Additional analyses of products sent to the Brazilian Federal Police identified both counterfeit and substandard materials and also documented undeclared anabolic steroids in some analyzed dietary supplements.^18^^,^^19^ Together, these findings reinforce the public health relevance of the underground AAS market in Brazil.^17^, ^18^, ^19^

## Polypharmacy and ancillary drug use

Polypharmacy, or the concurrent use of multiple drugs, is a defining feature of AAS abuse, employed to enhance effects, mitigate side effects, or hasten recovery.^15^ Users commonly combine AAS with anti-estrogens, such as tamoxifen, clomiphene citrate, or aromatase inhibitors, to counteract side effects such as gynecomastia or as part of post-cycle therapy intended to stimulate endogenous testosterone production.^15^^,^^16^

Additionally, AAS are frequently stacked with other Performance- and Image-Enhancing Drugs (PIEDs), such as human growth hormone and clenbuterol.^14^^,^^15^ In the HAARLEM cohort, 21% of users reported concurrent use of human growth hormone and 19% reported use of clenbuterol.^14^ This complex polypharmacy increases the unpredictability of toxicological outcomes and drug-drug interactions.^15^

### *Evidence of health consequences*

Chronic exposure to supraphysiologic doses of AAS results in widespread and severe adverse health consequences across multiple organ systems.^2^^,^^15^

### *Cardiovascular consequences*

AAS abuse is strongly linked to cardiovascular toxicity.^20^^,^^21^ Long-term use has been associated with myocardial dysfunction, reduced left ventricular systolic and diastolic function, accelerated coronary atherosclerosis, and increased cardiovascular morbidity.^20^^,^^21^ Population-based data also suggest increased risks of serious cardiovascular events and premature mortality among users.^24^

### *Hepatic and metabolic consequences*

AAS use is closely associated with hepatotoxicity, particularly with orally administered 17α-alkylated steroids.^2^^,^^22^ Chronic abuse can cause hepatic disorders including cholestasis, peliosis hepatis, hepatic adenomas, and even hepatocellular carcinoma.^2^^,^^22^ Metabolically, AAS misuse may promote dyslipidemia, insulin resistance, and visceral fat accumulation.^20^

### *Reproductive and endocrine consequences*

Suppression of the hypothalamic-pituitary-testicular axis is a near-universal consequence of exogenous AAS use.^2^^,^^15^ This suppression may lead to testicular atrophy, infertility, impaired spermatogenesis, gynecomastia, acne, androgenic alopecia, and prolonged hypogonadism after discontinuation.^15^^,^^23^

### *Neuropsychiatric consequences*

AAS abuse is associated with important psychiatric symptoms.^15^^,^^16^ These include mood disturbances such as mania or hypomania during active use and depressive symptoms or depression during withdrawal.^15^^,^^16^ Increased aggression and violent behavior have also been described, and AAS use may coexist with polysubstance use and dependence-related patterns.^15^^,^^16^ In addition, AAS users appear to face an increased risk of premature mortality.^24^

### *Hematologic effects*

Erythrocytosis is among the most frequent adverse effects of exogenous testosterone exposure and represents a clinically relevant safety concern because marked hematocrit elevation may increase blood viscosity and thrombotic risk.^4^^,^^15^ Accordingly, guidelines for medically supervised TRT recommend baseline and periodic hematocrit monitoring, with dose adjustment, temporary interruption, or investigation of contributing factors when thresholds are exceeded.^4^ In the setting of supraphysiologic AAS abuse, this hematologic risk is likely to be amplified.^15^

### *Prostatic effects*

Experimental studies suggest that supraphysiologic doses of AAS may induce structural changes in the prostate, including increased gland weight and volume, epithelial hyperplasia, and stromal remodeling.^25^ Clinical evidence in humans, however, remains limited and indirect. Available data from studies on testosterone therapy cohorts suggest that exogenous androgens may influence prostate biology and lower urinary tract function, but these findings do not directly address men abusing supraphysiologic AAS doses.^6^^,^^7^^,^^26^^,^^27^ Therefore, although a biologically plausible association exists, current human evidence remains insufficient to support firm conclusions regarding benign prostatic hyperplasia, lower urinary tract symptoms, or prostate cancer specifically in this setting.^6^^,^^7^^,^^15^^,^^26^^,^^27^

### *Sexual effects*

The relationship between AAS exposure and sexual function is complex and often phase-dependent. Some users report transient increases in libido during active cycles, whereas erectile dysfunction and decreased sexual desire are frequently described during withdrawal or in the context of prolonged hypothalamic-pituitary-gonadal suppression. Persistent hypogonadism, infertility, and sustained sexual dysfunction may occur in a subset of users, underscoring the need for clinical recognition and longitudinal follow-up in this population.^1^^,^^15^^,^^23^

## Public health implications

The severe morbidity and premature mortality linked to AAS use establish this practice as an important public health issue.^8^^,^^10^^,^^24^ Given the hidden nature of the problem, particularly among recreational weightlifters and gym users who often do not disclose use to physicians, clinicians should maintain a high index of suspicion.^2^^,^^13^^,^^15^ AAS exposure should be considered in the differential diagnosis of otherwise unexplained cardiovascular, endocrine, reproductive, hepatic, sexual, or psychiatric complaints in young or middle-aged individuals.^15^^,^^20^^,^^23^^,^^24^

In parallel with prevention, clinicians should be prepared to identify and support individuals already using AAS. A non-judgmental history should include cycling and stacking practices, co-use of other substances, and likely black-market exposure. Evaluation should focus on cardiovascular, hepatic, endocrine, reproductive, hematologic, and psychiatric complications, with referral pathways to endocrinology/andrology, cardiology, hepatology, and psychiatry/psychology when needed. Abrupt cessation may precipitate severe withdrawal hypogonadism, depression, and suicidality; therefore, decisions regarding discontinuation and subsequent management must be individualized, ideally within a multidisciplinary team. Monitoring and managing persistent hypogonadism, infertility, and mood disorders are central, with structured follow-up and access to psychological support to reduce relapse risk. In practice, many users are ambivalent about stopping; in such cases, harm-reduction strategies may serve as intermediate steps, while clearly emphasizing that ongoing supraphysiologic exposure remains hazardous. AAS dependence should be approached as a chronic condition requiring sustained counseling, mental health support, and medical follow-up rather than a one-off intervention.^1^^,^^2^^,^^15^^,^^16^

From a public health perspective, addressing this problem requires systematic intervention. Public awareness campaigns and educational efforts must target the severe health consequences of AAS abuse.^2^^,^^8^^,^^17^ Furthermore, enforcement of regulations and laws against the illicit supply chain is essential, particularly given the high incidence of counterfeit and substandard products documented in Brazil.^17^, ^18^, ^19^

## Consequences for women's health

Although testosterone plays a physiological role in female health, influencing libido, mood, bone density, and cognitive function, its clinical use in women requires caution. Current evidence does not support the widespread use of testosterone therapy in women, particularly when administered through compounded formulations such as subdermal pellets. A critical review of hormonal pellets concluded that the quality of evidence supporting testosterone use in women is very low, with studies marked by methodological limitations, small sample sizes, inconsistent findings, and a lack of long-term safety data. Supraphysiologic levels frequently observed with pellet use increase the risk of androgenic adverse effects, including acne, hirsutism, voice changes, and mood disturbances, without demonstrated benefit for most gynecological or non-medical indications. Although androgen abuse among women seeking aesthetic or performance enhancement is less prevalent than in men, it is increasingly recognized and carries unique, and sometimes irreversible, risks that warrant specific clinical attention and public health vigilance.^9^^,^^28^

## Limitations

This manuscript has some inherent limitations. First, much of the available epidemiological data on AAS use relies on self-reported information, which is subject to recall bias and underreporting, particularly given the illicit nature of these substances. Second, prevalence estimates vary widely because of heterogeneity in study design, sampled populations, and regional characteristics, limiting direct comparability between studies. Third, although this manuscript integrates Brazilian data whenever available, several mechanistic and long-term outcome studies derive from international cohorts, which may not fully reflect local sociocultural and regulatory contexts. Finally, as an editorial and clinical warning manuscript, this work does not aim to provide a systematic review or meta-analysis, but rather to contextualize existing evidence and highlight public health implications, which should be further explored in prospective, population-based studies.

## Conclusion

Testosterone and other AAS abuse constitute a growing public health challenge in Brazil, driven by appearance-focused motivation and fueled by an unregulated black market supplying high-dose, often counterfeit products. The severe and long-term consequences, affecting cardiovascular, hepatic, reproductive, endocrine, hematologic, sexual, neuropsychiatric, and women's health domains, demand a cohesive response from the medical and regulatory communities. Clinicians should educate patients, actively screen at-risk individuals, and contribute to targeted multidisciplinary interventions. Concerted efforts focused on effective public health campaigns, stronger enforcement of regulations and laws against illicit suppliers, and the establishment of clinical support for individuals struggling with AAS dependence are critical next steps to reduce the health burden of this growing problem.

## Funding

This research did not receive any specific grant from funding agencies in the public, commercial, or not-for-profit sectors.

## Declaration of competing interest

The authors declare no conflicts of interest.

## Data Availability

The datasets generated and/or analyzed during the current study are available from the corresponding author upon reasonable request.
